# Association of sleep network functional connectivity with hyperexcitability and cognition in Alzheimer’s disease

**DOI:** 10.1002/alz.089546

**Published:** 2025-01-09

**Authors:** Sebastian Moguilner, Courtney Berezuk, Alex C Bender, Kyle R Pellerin, Stephen Gomperts, Sydney Cash, Rani A Sarkis, Alice D Lam

**Affiliations:** ^1^ Massachusetts General Hospital, Boston, MA USA; ^2^ Harvard Medical School, Boston, MA USA; ^3^ Brigham and Women's Hospital, Boston, MA USA

## Abstract

**Background:**

Sleep disturbances are common in Alzheimer's disease (AD) and occur at early stages. Hyperexcitability also arises during sleep and can lead to epileptiform activity and seizures that impact memory consolidation. The underlying mechanisms of sleep disturbances and hyperexcitability in AD pathology remain unclear but are likely associated with changes in brain networks and altered functional connectivity (FC).

**Method:**

Using ambulatory scalp EEG recordings, we examined 33 cognitively normal healthy older adult controls (HC), 36 individuals with early clinical stages of AD without history or risk factors for epilepsy (AD‐NoEp), and 14 individuals with early clinical stages of AD who developed epilepsy related to Alzheimer's (AD‐Ep). The analysis involved analyzing FC measures such as amplitude envelope correlation with leakage correction, coherence‐based measures, and graph theory to examine FC across sleep‐wake stages, frequency bands, and its associations with cognitive performance.

**Result:**

We found specific FC changes in Alzheimer's during sleep stages and frequency bands, including increased gamma connectivity during N2 sleep (N2‐gamma), increased delta connectivity during REM‐delta, and decreased alpha connectivity in N2‐alpha and REM‐alpha. AD‐Ep showed a distinct network signature compared to AD‐NoEp, with changes in sleep‐stage and frequency band combinations not observed in AD‐NoEp and reversal of FC changes associated with AD‐NoEp. Specific measures (N2‐gamma, REM‐alpha, Awake‐theta, and Awake‐delta connectivity) were linked to longitudinal global cognitive decline in AD. Machine learning algorithms using combinations of awake and asleep FC features accurately distinguished AD‐NoEp from HC (AUCroc = 0.905) and AD‐Ep from AD‐NoEp (AUCroc = 0.881). Moroever, we identified "fast cognitive decliners" among AD participants with high classification performance (AUCroc = 0.865).

**Conclusion:**

The results highlight specific changes in sleep network functional connectivity in AD, offering insights for disease monitoring and finding therapeutic targets.

